# Sex-differences in Mothers' own milk and neurodevelopmental outcomes in preterm infants

**DOI:** 10.3389/fped.2025.1523952

**Published:** 2025-03-28

**Authors:** Tingting Zhao, Aolan Li, Xiaolin Chang, Wanli Xu, Tyler Quinn, Jie Chen, Adam P. Matson, Ming-Hui Chen, Sarah N. Taylor, Xiaomei Cong

**Affiliations:** ^1^ School of Nursing, Yale University, Orange, CT, United States; ^2^ School of Nursing, Columbia University, New York, NY, United States; ^3^ Department of Statistics, University of Connecticut, Storrs, CT, United States; ^4^ School of Nursing, University of Connecticut, Storrs, CT, United States; ^5^ College of Nursing, Florida State University, Tallahassee, FL, United States; ^6^ Division of Neonatology, Connecticut Children’s Medical Center, Hartford, CT, United States; ^7^ Department of Pediatrics, University of Connecticut School of Medicine, Farmington, CT, United States; ^8^ Department of Pediatrics, Yale University, New Haven, CT, United States

**Keywords:** preterm, neurodevelopment, feeding, sex, breastmilk

## Abstract

**Objective:**

To identify sex-specific feeding patterns and associations with growth and neurodevelopment in preterm infants during NICU through 2 years of corrected age (CA).

**Methods:**

A cohort study was conducted with 216 preterm infants (gestational age 28 0/7 to 32 0/7 weeks). Daily feeding regimens, including mother's own milk (MOM), human donor milk, and formula; daily growth; acute and chronic pain/stress were documented during NICU. NICU Network Neurobehavioral Scale (NNNS) (36 to 38 postmenstrual age), and Bayley Scales of Infant and Toddler Development (Bayley) Edition III (1 and 2 years of CA) were measured.

**Results:**

Between week 9 to 16 after birth, only females showed a positive association between growth z-score and proportion of MOM intake before week 8 (*p* < 0.05). Sex-differentiated associations between MOM and stress were observed (*p* < 0.05). MOM proportion was positively correlated with language or cognitive scores at 2 years of CA in females (*p* = 0.01), this correlation not evident in males.

**Conclusions:**

We discovered a sex-specific “window of opportunity” for feeding, growth and risk predictors for neurodevelopment up to 2 years of CA. These insights may inform development of tailored feeding regimens, potentially mitigating growth and development differences observed between males and females.

## Introduction

Even with advances in neonatal intensive care, preterm infants [<37 weeks of gestational age (GA)] still face tremendous challenges to surviving and thriving. Notably, males born preterm are consistently observed to have a higher risk of perinatal complications, with worse mortality and morbidity (1–4), and neurodevelopmental impairments (5–7) than females. The etiology of the “sex bias” in preterm growth and health remains unclear and likely multifactorial (4). Sex variation in nutritional need is increasingly recognized as playing a significant role in the growth and health outcomes of preterm infants (8). Emerging studies show sex-specific responses to nutritional intake (8–13), suggesting potential sex-based nutrition requirements in early life for optimal development.

Mother's own milk (MOM) is ideal for preterm infants' growth, immunity, and neurodevelopment (14, 15). The amount and composition of MOM has been found dynamically adjusted to support the infants' nutritional requirements along with their developmental phase (16). Emerging research shows the interplay between the MOM characteristics and infant sex for tailoring sex-specific needs of the offspring (16). While mothers of term male infants are more likely to produce milk with higher levels of energy, free amino acids, total lipids, phospholipids, and gangliosides, which are significantly associated with infants' neurobehavioral development (16–20). There is limited evidence of sex-specific response to feeding in preterm infants. Moreover, it is challenging to conclude the impact of sex-specific MOM on the growth and development of preterm infants. This challenge arises from the intricate difficulties faced by mothers in terms of breastfeeding and providing MOM for their infants (15, 21).

Our study aims to examine the associations between sex-specific responses to feeding, especially MOM intake, and the growth and neurodevelopment of preterm infants from NICU up to 2 years of corrected age (CA). We hypothesize that male and female preterm infants have distinct feeding needs at the early stage of their lives, which contribute to their growth and neurodevelopmental outcomes. Therefore, we used linear mixed models to evaluate the significance of factors including feeding and clinical data, in predicting the growth and neurodevelopmental outcomes in both male and female infants. Further, we applied SHapley Additive exPlanations (SHAP) to determine the importance of risk factors on predicting neurodevelopmental outcomes.

## Methods

### Study design and participants

We conducted a cohort study to investigate the associations of feeding regimens with growth and neurodevelopment outcomes in male and female infants from NICU up to 2 years of CA. The study was approved by the IRBs at the study institutes and the medical center in Northeastern United States. A total of 216 preterm infants were recruited between 2017 and 2022 at two affiliated NICU sites. **Inclusion criteria** were preterm infants: (1) born at 28 0/7 to 32 0/7 weeks of GA, and (2) received consent from parents who were ≥18 years old. **Exclusion criteria** were infants who have: (1) known congenital or chromosomal abnormalities; (2) severe periventricular/intraventricular hemorrhage (≥ grade III); (3) undergone surgery; and/or (4) illicit substances exposure history during pregnancy.

### Demographic and health characteristics

Infants' demographic and health information were collected. The Score for Neonatal Acute Physiology with Perinatal Extension-II (SNAPEII) (22) and treatment with antibiotics before or after the first 3 days after birth were also measured.

### Feeding regimens

Infants' daily feeding information was collected by research nurses, including the frequency of infant fed by MOM, fortified human donor milk (HDM), and/or fortified formula during their NICU study. Based on the NICU feeding protocol, all infants with a birth weight <1,800 grams and/or birth GA < 32 weeks were eligible to receive HDM when MOM was unavailable. The transition from HDM to formula occurred at 34 weeks of GA. The total number of frequency of feedings (up to 8 times per day) and quantity of MOM, HDM, and formula given to infants during each shift and daily were collected. The daily proportion of MOM was then calculated given its frequency over the total number of daily feedings. Given that a significant number of preterm infants received a combination of feeds, we classified feeding types using a 70% threshold for both frequency and amount for a specific period (23, 24). For example, if an infant was predominantly fed with MOM for over 70% of the total feeding during week 1, the infant was categorized as MOM for that week. Similarly, if an infant received a mixture of formula and MOM, with the combination accounting for more than 70% of the feeds during week 2, the coding would be MOM + formula for week 2.

### Early life painful/stressful experiences during the NICU stay

Infants' acute and chronic painful/stressful events experienced during NICU based on the revised NICU Infant Stressor Scale (NISS*)* were documented in the REDCap by research nurses (25, 26). Acute and chronic painful/stressful procedures were categorized into five domains, ranging from 1 (not painful/stressful) to 5 (signifying extremely painful/stressful). The average daily pain/stress for the first 28 days after birth was computed by adding the daily frequencies (for acute) and hours (for chronic) of pain/stress events for all four weeks, then dividing by 28 days.

### Growth measurements

From birth to 16 weeks of postnatal age, infants' daily or weekly growth parameters including weight, length, and head circumferences were documented by research nurses. Infants' weight was assessed using an electronic scale with a capacity of 20 kilograms and an accuracy within 10 grams. The scale was calibrated every 2–3 months. Body length was measured using the Shorr Infant/Child Height Measuring Board, subject to monthly inspections for accuracy, with measurements recorded to the nearest 0.1 cm. Head circumference was gauged with a disposable tape accurate to the nearest 0.1 cm. Measures were taken 3 times, assuring agreement within 30 grams and 1 cm. If the established limit was surpassed, a fourth measurement was obtained, and the three closest measurements were utilized.

### Neurobehavior responses and neurodevelopment measurements

NICU Neonatal Neurobehavioral Scale (NNNS) was assessed when infants reached their 36–38 weeks post-menstrual age (PMA) (27). NNNS includes 115 items which are categorized into 13 summary scores for evaluating habituation, attention, arousal, self-regulation, handling, quality of movement, excitability, lethargy, non-optimal reflexes, asymmetric reflexes, hypertonicity, hypotonicity, and stress/abstinence. Our study focused on stress/abstinence (NSTRESS), quality of movement (NQMOVE), and arousal (NAROUSAL). Lower NSTRESS and NAROUSAL subscale scores, and higher NQMOVE subscale scores indicate better neurodevelopmental outcomes.

Bayley Scales of Infant and Toddler Development (Bayley) Edition III (28) was used to assess children's cognitive, language, and motor skills at 1 and 2 years of CA. A higher score within each domain or a total score across all domains indicates better neurodevelopmental outcomes for infants. This is the most widely used, valid, and reliable assessment tool for developmental assessment of preterm infants (29, 30). Certified research nurses administered the NNNS assessment and formally accredited neonatologists conducted the Bayley assessment. Evaluators were blinded to infant feeding exposure.

### Statistical analysis

The analysis focused on assessing the effects of various exposure variables including MOM proportion, total feeding volume, sex, and the interaction of MOM proportion and sex, on infant growth and development. We evaluated infant growth outcomes (weight z-score), NNNS, and Bayley scores at 1 and 2 years of CA, as the primary outcomes in our study. To examine the association between exposure variables and weight z-score, we employed linear mixed models (LMM) (31). To analyze NNNS and Bayley scores, we employed linear models to examine their direct association with exposure, as well as adjusted for confounders including race, ethnicity, birth GA, weight and length, acute and chronic pain experience, SNAPPEII, NISS, and delivery mode. This adjustment is critical for isolating the effects of key exposure variables on outcomes. Furthermore, to identify the most influential factors affecting NNNS and Bayley scores and understand their degree of influence, we employed XGBoost (32) for feature importance analysis. This was complemented by the SHapley Additive exPlanations (SHAP) (33) to determine the importance of the risk factors in predicting neurodevelopmental outcomes. Additionally, we utilized growth curve modeling (34) to construct infant growth trajectory scores, labeled as 's' representing the growth slope. All analyzes were performed using R version 4.2.0 and *p* < 0.05 was considered statistically significant.

## Results

### Preterm infant and maternal demographic and clinical characteristics

Our study recruited a total of 216 infants with a majority of males (57.9%), White (67.6%), and non-Hispanics (70.5%). The infants were born at a median GA of 28.6 weeks (IQR, 26.6–30.1), with a median birth weight of 1052.5 gram (IQR, 815.0–1322.5), a median SNAPPEII score of 21.0 (IQR, 10.0–34.0), and treated with antibiotics during first 3 days after birth (94%). In addition, male infants exhibited higher birth weight, body length, and head circumference (*p* < 0.05), compared to female infants (see Table 1), while there was no significant difference in birth GA between two groups. The median age of mothers of preterm infants was 31.0 years (IQR, 27.0–35.0), and 54.2% of them were married (see Supplementary Table S1).

**Table 1:** Infant demographic characteristics (*N* = 216).

Variables	Total (*N* = 216)	Female (*n* = 91)	Male (*n* = 125)	*p*-value
Race				
White	146 (67.6)	53 (58.2)	93 (74.4)	0.11
Black	51 (23.6)	27 (29.7)	24 (19.2)	
Other	19 (8.8)	11 (12.1)	8 (6.4)	
Ethnicity				
Hispanic	54 (25.0)	21 (23.1)	33 (26.4)	0.69
Non-Hispanic	162 (75.0)	70 (76.9)	92 (73.6)	
Delivery				
C-section	152 (70.4)	67 (73.6)	85 (68.0)	0.46
Vaginal	64 (29.6)	24 (26.4)	40 (32.0)	
PPROM				
Yes	48 (22.2)	20 (22.0)	28 (22.4)	1.0
Antibiotic used first 3 days				
Yes	203 (94.0)	89 (97.8)	114 (91.2)	0.09
Antibiotic used after 3 days				
Yes	31 (14.4)	15 (16.5)	16 (12.8)	0.57
	Median (IQR)	Median (IQR)	Median (IQR)	*p*-value
Birth GA (week)	28.6 (26.6, 30.1)	28.3 (26.6, 29.7)	28.7 (26.6, 30.4)	0.24
Birth weight (g)	1052.5 (815.0, 1322.5)	930.0 (785.0, 1240.0)	1110.0 (865.0, 1375.0)	0.01
Birth body length (cm)	36.3 (34.0, 39.5)	35.6 (34.0, 39.0)	37.0 (34.0, 40.0)	0.03
Birth HC (cm)	25.5 (23.5, 27.5)	25.0 (23.2, 26.5)	26.0 (23.9, 28.0)	0.02
SNAPPEII	21.0 (10.0, 34.0)	24.0 (14.0, 35.0)	19.0 (9.0, 33.0)	0.07

Abbreviation: PPROM, Preterm premature rupture of membranes; HC, head circumference; GA, gestational age; SNAPEII, score for neonatal acute physiology with perinatal extension-II.

### Feeding intakes during NICU hospitalization

**Figure 1:**
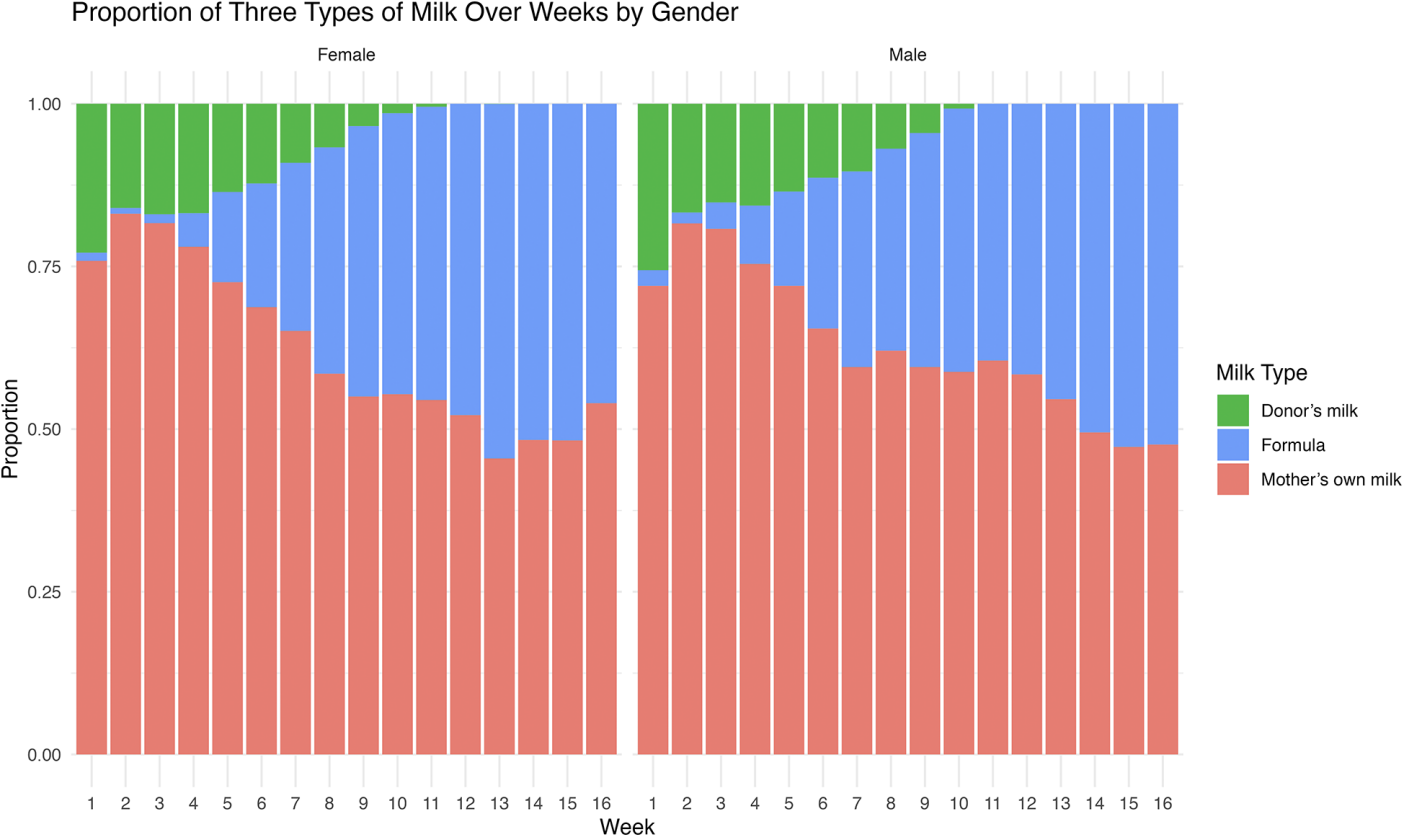
The weekly proportion of feeding regimen in the NICU. The weekly proportion of feeding regimens, including mother's own milk, Donor's milk, and Formula, is compared between males and females throughout their NICU stay from week 1 to 16.

We observed a consistent, although not statistically significant, increase in total feeding amount and variations in the proportion of MOM, HDM, and formula, during postnatal weeks 1 to 16 for both male and female infants (Figure 1, Supplementary Figure S1). Initially, there was an increase in the proportion of MOM during the first 3 weeks of the NICU stay. However, the proportion of MOM and HDM feeding decreased over time, in contrast, the proportion of formula increased. Specifically, from the 2nd postnatal week to the 8th, the proportion of MOM decreased from 81.6% to 62.1% in males, and from 83.1% to 58.5% in females, there was no significant difference in the proportions of feeds between sexes (Table 2).

**Table 2:** Proportion of feeding types for male and female preterm infants at postnatal week 2 and week 8.

Variables	Total % (*N* = 215)	Female % (*n* = 91)	Male % (*n* = 124)	*p*-value
Week 2				
MOM	82.3 (33.0)	83.1 (32.2)	81.6 (33.7)	0.75
Human donor milk	16.4 (31.6)	16 (31.2)	16.7 (32.0)	0.87
Formula	1.3 (10.5)	0.9 (6.1)	1.7 (12.9)	0.55
Variables	Total (*N* = 179)	Female (*n* = 86)	Male (*n* = 93)	*p*-value
Week 8				
MOM	60.4 (44.3)	58.5 (45.3)	62.1 (43.5)	0.60
Human donor's milk	6.8 (22.7)	6.7 (23.4)	6.9 (22.3)	0.95
Formula	32.8 (41.4)	34.8 (43.1)	30.9 (40.0)	0.53

### Sex-specific feeding and growth

**Figure 2:**
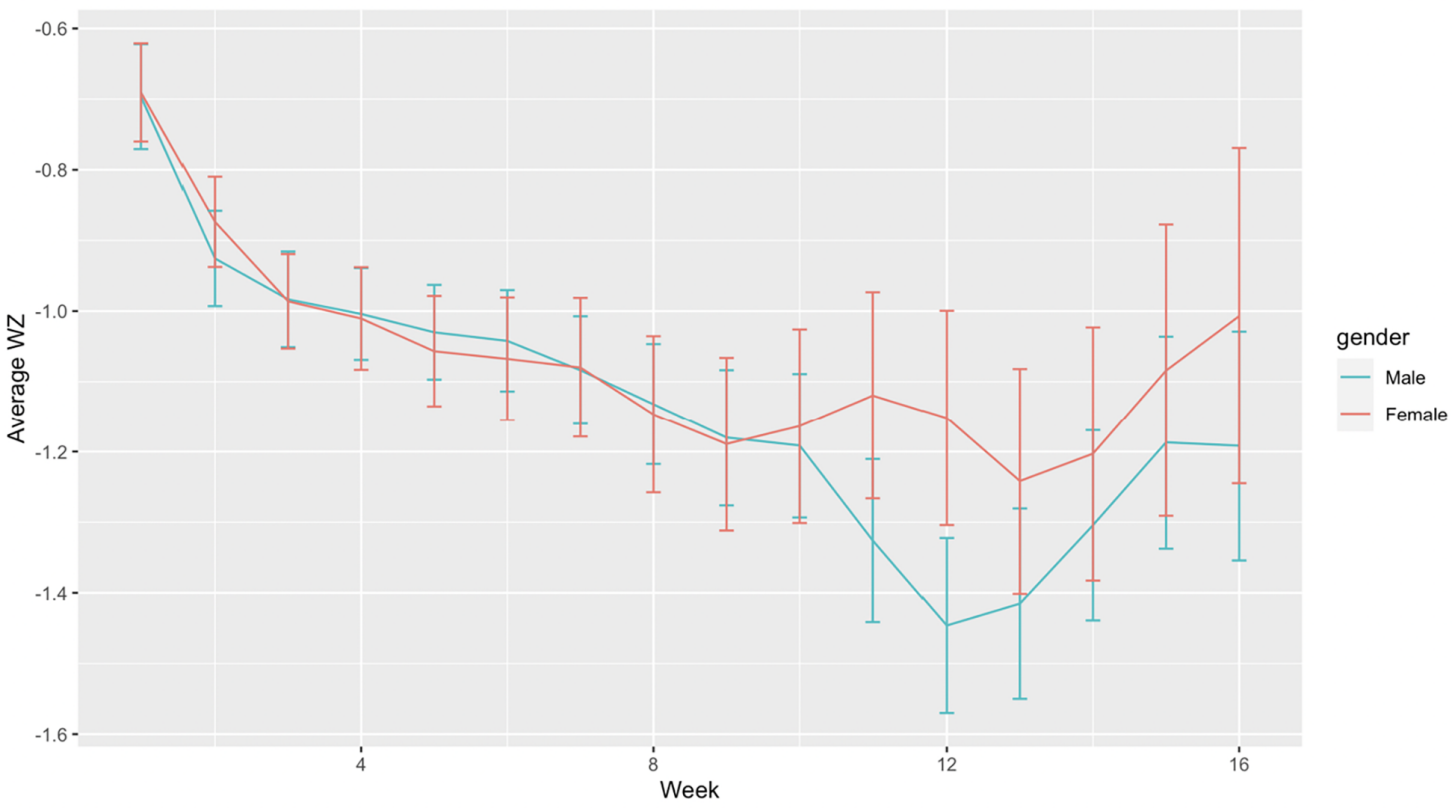
Weekly mean growth trajectory by sex.

Although both males and females showed similar patterns in growth, females had a slightly higher average growth z-score compared to males between week 9 to 16 postnatal age, (Figure 2), despite males having a significantly higher birth weight (Table 1) and receiving similar feeding proportions between week 2 to 8 (Figure 1). The proportion of MOM intake and birth weight showed a positive correlation with the growth z-score, while SNAPPEII was negatively correlated with the growth z-score in both males and females (all *p* < 0.05). From weeks 1 to 8, total feeding amount was found to be positively correlated with the growth z-score in female infants (*p* < 0.05), while no significant relationship was observed in males (Supplementary Table S2). Furthermore, the growth of females from 9 to 16 weeks was positively associated with a higher proportion of MOM intake during the first 8 weeks (*p* < 0.05), whereas the growth of males was associated with the total feeding amount at that time (*p* < 0.05) (Supplementary Table S2).

### Proportion of MOM intake and neurobehavioral responses in the NICU

After adjusting for confounders such as birth weight, length, GA, early life painful and stressful experience, and delivery types in the regression models, we observed the association of sex-dependent patterns in how the feeding intake during the first 8 postnatal weeks with the neurobehavioral responses (NSTRESS and NAROUSAL) at NICU. The association between MOM intake and NSTRESS differed by sex, with a quadratic relationship between MOM intake and NSTRESS in females compared to males (1st order coefficient *b* = 0.02, *p* = 0.9; 2nd order coefficient *b* = −0.5, *p* < 0.05) (Supplementary Figure S2). Females consuming more than 1,000 cc of MOM per week had significantly lower NAROUSAL compared to males (coefficient *b* = −0.5, *p* < 0.05) (Supplementary Figure S3).

### Proportion of MOM intake and neurodevelopment at 1- and 2-year follow-up

Unless specified otherwise in our findings, the MOM proportion refers to the period during the first 8 postnatal weeks. At the 1-year follow-up, no significant correlations were observed between MOM and Bayley scores. At 2 years of age, these associations differed by sex. In females, the proportion of MOM intake was positively correlated with cognitive scores (coefficient *b* = 15.8, *p* = 0.03) and language (coefficient *b* = 18.77, *p* = 0.01), whereas such correlation was not observed in males (Supplementary Table S3).

### SHAP predicting sex-differentiated risk factors of early neurobehavior (NICU) and later neurodevelopment (1 and 2 years of age)

**Figure 3:**
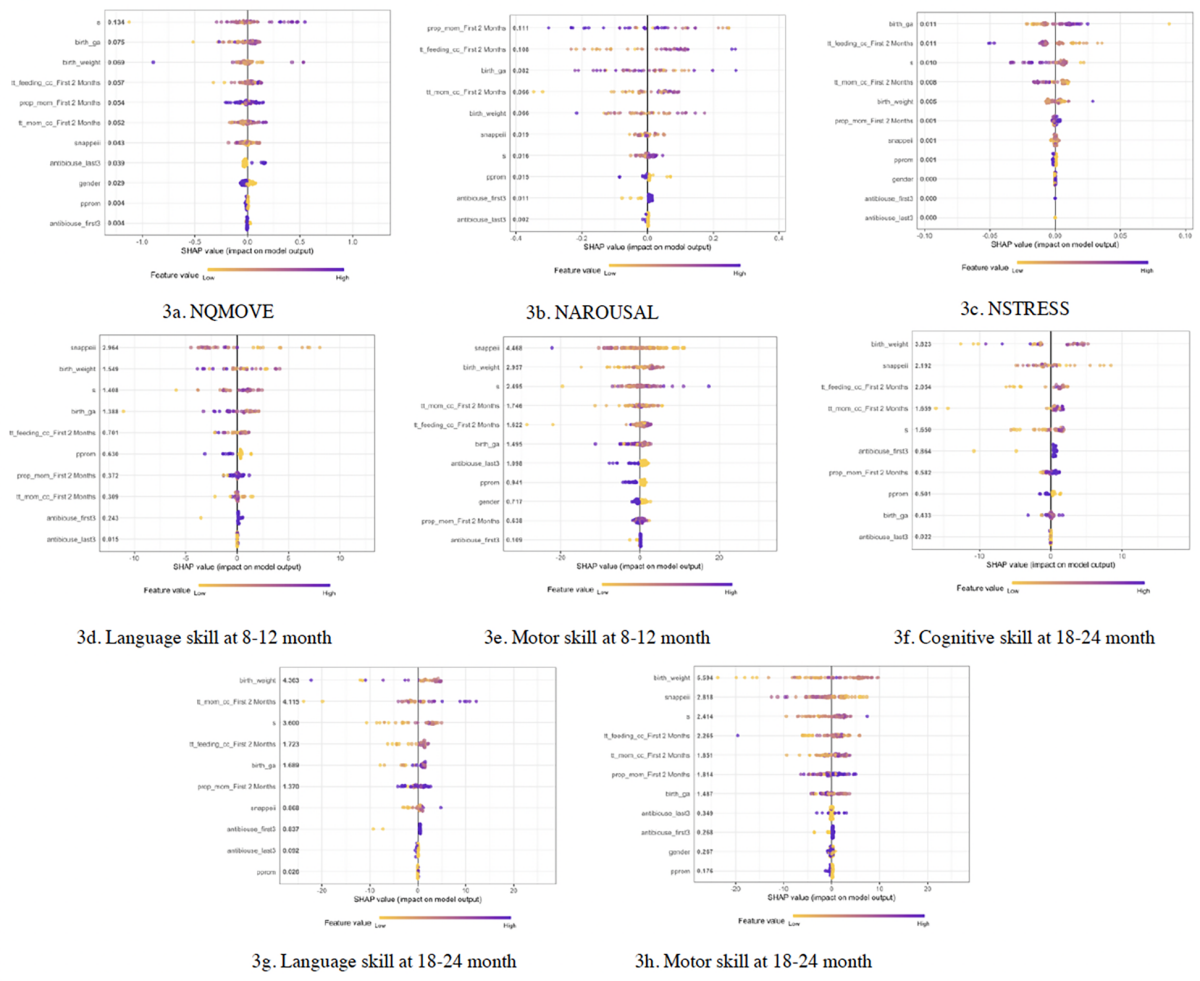
SHAP predicting risk factors for neurobehavioral and neurodevelopmental outcomes in infants. **(a–c)** Risk factors predicting NQMOVE, NAROUSAL, and NSTRESS. **(d,e)** Risk factors predicting Language and Motor skills at 8 to 12 months of corrected age (CA). **(f–h)** Risk factors predicting Cognitive, Language, and Motor skills at 18 to 24 months of CA.

Overall, birth GA, birth weight, SNAPPEII, total feeding amount, total amount of MOM intake, proportion of MOM, and growth during 1 to 8 postnatal weeks emerged as significant predictive factors influencing early-life neurobehavioral outcomes and later neurodevelopment in both males and females (Figure 3). However, the relative importance of each specific risk factor varied between sexes. For males, the top 5 risk predictors, in order of importance, were birth weight, total MOM, SNAPPEII, growth slope, and birth gestational age. In females, the top 5 risk predictors, in order of importance, were growth slope, birth weight, birth GA, total feeding amount, and total MOM.

## Discussion

Sex differences become apparent early in development and persist into later stages in preterm infants (35), however, the underlying mechanisms remain poorly understood. Feeding regimen has emerged as a potential factor that may influence the sex-differentiated growth in infants (36). Therefore, our study investigated sex-specific response, such as early life growth (1 to 16 postnatal weeks), to different feeding regimens. This timeframe aligns with the “window of opportunity” identified in other studies, playing a critical role in shaping infants' health outcomes (37). We also consider week 1 to 8 as “early time” and week 9 to 16 as “later time” to look at even closer to the sex-varied growth during this window of opportunity. We observed the association of both total feeding amount and proportion of MOM with the growth of males and females is sensitive to their postnatal age. MOM proportion during postnatal weeks 1–8 is associated with the growth of both males and females. This association persists in females beyond 8 postnatal weeks, but not in males. In addition, early total feeding is also associated with females' growth but not for males. This could be explained by the different responses of females and males to the nutritional composition of feeding (38). Personalizing essential nutrient intake may help reduce gender differences. Furthermore, sex-differentiated breastmilk components, such as oligosaccharides (HMOs) (39), mineral (40), cortisol (41), and protein profiles (42) amplify this distinction. This disparity was referred to as “male disadvantage” (43). We suspected that females may benefit more from early MOM, with this benefit continuing as they age. This promotes essential nutrient intake and accelerated growth. This consideration arises while recognizing the growth of males might be “delayed” due to reduced MOM intake in their later life, or perhaps they require different nutrients not provided by MOM. Additionally, other components of breast milk, such as 8- hydroxy-2′-deoxyquanosine (8-OHdG), histone deacetylase sirtuins (SIRT3) and serotonin (5-HT) have been associated with inflammation and oxidative stress induced by premature and/or cesarian delivery (44). However, the sex specific effects of these breast milk components on infant growth remain unknown (44). Further research is needed to explore the association between MOM components and infant growth and neurodevelopment.

The earliest indication of sex-differentiated post-birth growth is reflected in males having significantly higher birth weights than females (45). Our study aligns with this pattern; however, we also observed a decrease in body weight over time in male infants, which was consistent with the findings of other study (46). In our study, body weights in both males and females exhibited a significant post-birth increase, although the growth was relatively slower, as suggested by the trajectories of their weight z-scores, possibly due to their non-term equivalent age (47). It is noteworthy that post 9 weeks after birth, females' growth (z-score) was faster than males. Interestingly, females had a higher intake of mother's breastmilk before week 9, and a higher intake of formula post week 9 in comparison to males, potentially contributing to their accelerated catch-up in body weight post week 9 compared to males. Our finding aligns with other studies indicating that formula intake is associated with fast increased weight gain (48). Although six-month exclusive breastfeeding is the recommended gold standard for infants' nutrition (49), our study observed that preterm infants often encountered medical issues and/or lactation difficulties, leading to disrupted breast milk expression. Therefore, supplementing breastfeeding with alternatives like HDM and formula is recommended to ensure their well-being when MOM is limited (50).

Although preterm infants typically undergo catch-up growth after birth, this rapid weight gain could bridge the growth gaps between preterm and term infants (51); however, it does not necessarily eliminate the risk of neurodevelopment disorders (52–54). Our study observed that at 36 to 38 weeks of PMA, male infants demonstrated a higher level of fussing and crying during the assessment. In contrast, females showed a more mature neurobehavioral development if they received a higher amount of MOM before 8 postnatal weeks. This may be explained by the fact that earlier and higher MOM exposure boosts infants' brain structure (55) and connectivity (56). Further, whole brain volume increases in direct proportion to macronutrient and protein intake (57), however, a gender-specific scaling law exists, such that the cerebral cortex in males grows at a relatively slower rate compared to overall brain volume (58). Additionally, compared to females, resting neurophysiological network communication is more significantly altered in very preterm males, which further contribute to their disrupted cognitive outcomes later in childhood (at 8 years old) (59). Our study identified that the positive association between earlier and higher proportion of MOM exposure and better language and cognitive persist until the age of 2 in females. These findings were supported by other studies indicating the positive impact of earlier and longer breastfeeding on language ability performance and cognitive development (18, 60–62, 63).

To the best of our knowledge, this is the first time using SHAP, one of the Machine Learning methods, to determine the importance of total feeding amount, the quantity of MOM, and the proportion of MOM within 8 weeks postnatal in predicting neurodevelopmental outcomes in both males and females from the NICU until their 2 years of age. Other studies found birth complications such as intubation, durations of ventilatory support, length of hospital stay, and whether the infant was receiving breastmilk can also be used as predictors to predict cognitive abilities at 2 years of age (64). Consistent with other studies that have highlighted factors such as birth weight, GA, SNAPPEII, etc. (65), our analysis also identified these as predictors of cognitive and language development. Males in our study had relatively older birth GA and higher birth weight, but experienced delayed growth compared with females, these findings underscore the importance of recognizing that males with older birth GA, yet experiencing delayed weight gain, may be at risk for delayed neurobehavioral outcomes. Notably, these sex-differentiated neurodevelopmental outcomes should be interpreted with caution, as comparable sex differences have also been reported in term infants (66). Our future study will include both preterm and term infants to investigate the impact of preterm birth risk on sex-related neurodevelopmental outcomes.

Various practical barriers and challenges impact breast milk utilization and infant care, including adverse maternal social determinants of health such as lack of family support, availability of caregiver, and knowledge of breastfeeding (67), single mother (68), younger maternal age and lower education level (69), and the inability of extremely preterm infants to breastfeed (69). To improve infant health outcomes, multiple strategies should be considered. First, breast milk intake should be recognized as a key parameter in NICU protocols for monitoring and assessing preterm infant outcomes. Additionally, promoting breast milk intake alongside early intervention programs, such as developmental therapies for preterm infants, may further enhance neurological outcomes (70, 71).

## Strength and limitations

Large sample size with equally distributed sex and race subsamples, and subjects follow up from NICU until 1 and 2 years of CA providing a comprehensive understanding of infants' health outcomes. In addition, the SHAP analysis validated the results obtained from linear mixed models. This approach provided further insights into how each predictor influences the developmental outcomes in infants. However, the study has limitations. The composition such as neurotrophic factors, lipids, and proteins, or calories of MOM, HDM, formula, and fortification were not evaluated, which could offer insights into the biomechanisms underlying the clinical manifestations and sex-specific micronutrients and macronutrients. The duration of breast milk exposure is another factor we will consider in future studies to better understand its impact on infants' growth and neurodevelopment. Additionally, the study did not incorporate maternal social determinants of health, which could potentially broaden our understanding of the maternal factors influencing sex-specific feeding regimens in preterm infants.

## Conclusion and relevance

Sex-specific MOM intake, but not HDM and formula, was associated with infants' growth and neurodevelopmental outcomes. In addition, sex-specific “window of opportunity” for feeding, growth, and development in early life and sex-specific risk predictors for neurodevelopment from the NICU to 2 years of CA can inform the development of tailored, sex-specific feeding plans. Our study further emphasizes that early initiation of breastfeeding and exclusive breastfeeding beyond 8 weeks after birth are critical for infants' growth and neurodevelopment. To meet their nutritional requirements for growth, infants—especially males —should receive appropriate supplements. Individualized assessment and feeding regimens should be provided to preterm infants during their NICU stay.

## Data Availability

The data generated and/or analyzed during the current study are available from the corresponding author on reasonable request.
