# Supplementary material for: Sex-differences in Mothers' own milk and neurodevelopmental outcomes in preterm infants
Source: Front Pediatr. 2025 Mar 28;13:1523952. doi: 10.3389/fped.2025.1523952 (PMC11985779; doi:10.3389/fped.2025.1523952)
Supplement: Supplementary file 1 [file Datasheet1.pdf]

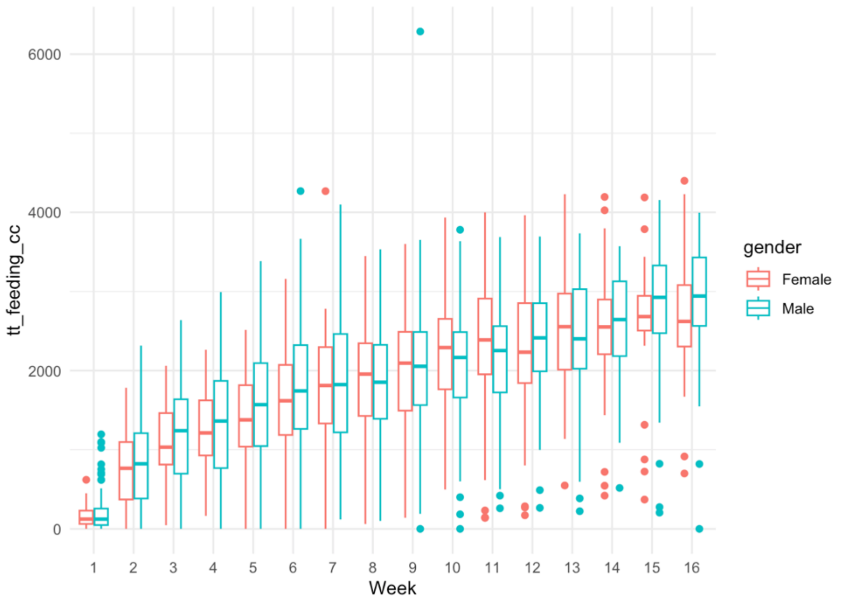

Supplementary Figure 1. Weekly total feeding amount. The weekly total amount of feeding intake comprising mothers' own milk (MOM), human donor milk, and formula, in male and female infants.

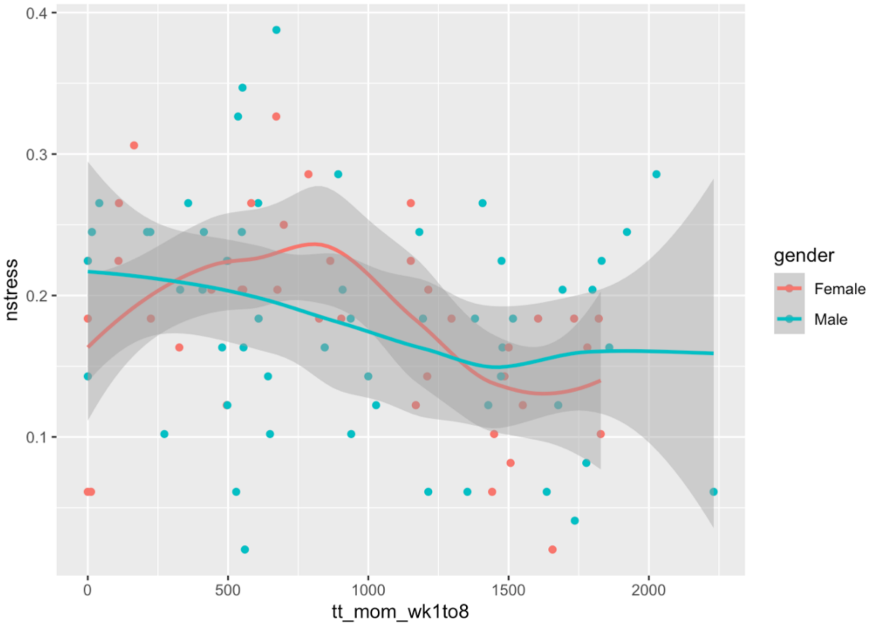

Supplementary Figure 2. Total mothers' own milk (MOM) intake between week 1 to 8 and NSTRESS in males and females.

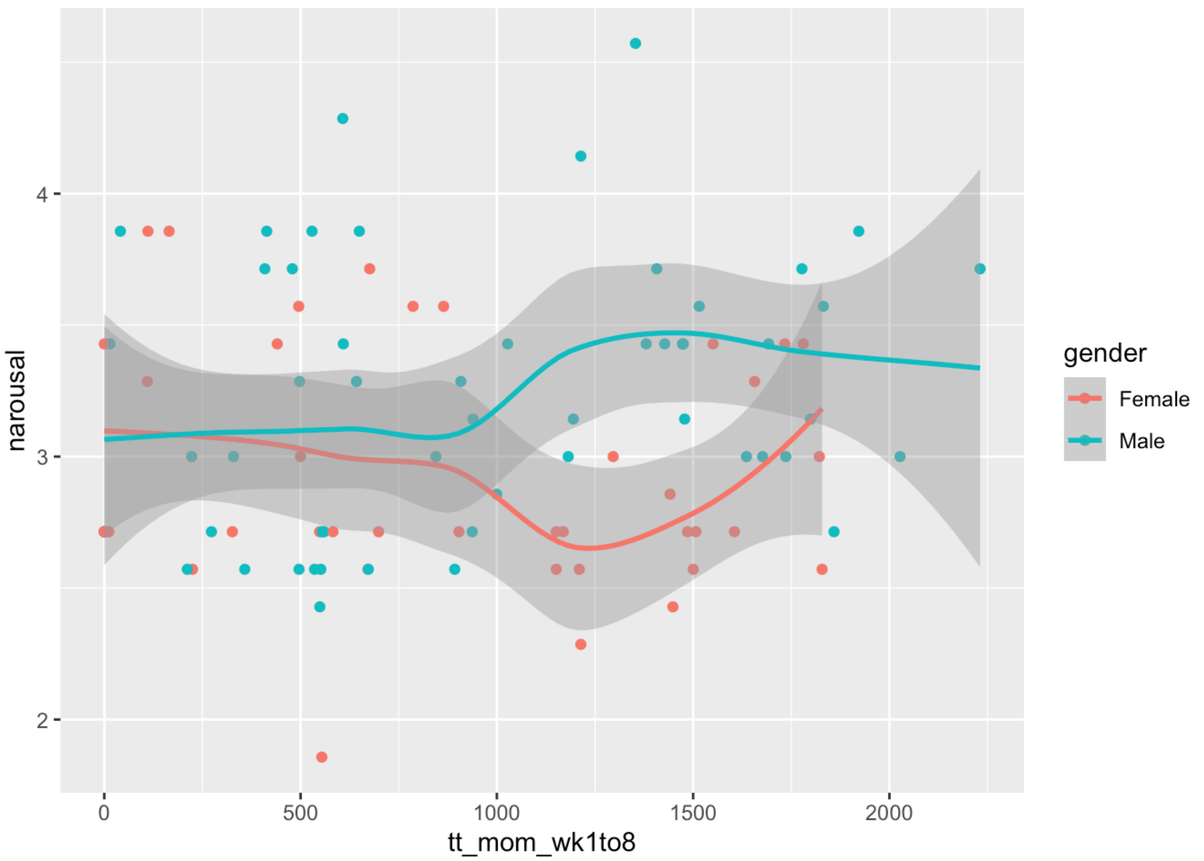

Supplementary Figure 3. Total mothers' own milk (MOM) intake between week 1 to 8 and NAROUSAL in males and females.

Supplementary Table 1. Maternal demographic characteristics (N=216)

Variables	Total (N=216)	Female (n=91)	Male (n=125)	p-value
Maternal race				
White	143 (66.2)	53 (58.2)	90 (72.0)	0.08
Black	52 (24.1)	27 (29.7)	25 (20.0)	
Other	21 (9.7)	11 (12.1)	10 (4.6)	
Ethnicity				
Hispanic	54 (25.0)	22 (24.2)	32 (25.6)	0.94
Non-Hispanic	162 (75.0)	69 (75.8)	93 (74.4)	
Maternal marital status				
Married	117 (54.2)	55 (60.4)	62 (49.6)	0.46
Single/divorced/separated	93 (43.1)	34 (37.4)	59 (47.2)	
Unknown	6 (2.8)	2 (2.2)	4 (3.2)	
	Median (IQR)	Median (IQR)	Median (IQR)	p-value
Maternal age	31.0 (27.0, 35.0)	31.0 (27.0, 34.0)	31.0 (26.5, 35.0)	0.75

Supplementary Table 2. Feeding factors and growth z-score in male and females during week 1 to 8 and week 9 to 16.

	Week 1 to 8	Week 1 to 8	Week 9 to 16	Week 9 to 16
	Proportion of MOM	Feeding total amount	Proportion of MOM	Feeding total amount
Males z-score	+0.11**	+0.03	+0.17	0.05*
Females z-score	+0.21***	+0.17***	+0.54*	-0.01

Note. + sign is the positive correlation, - sign is the negative correlation; *, p<0.05; **, p<0.01; ***, p<0.001; MOM, mother's own milk.

Supplementary Table 3. Proportion of MOM intake and neurodevelopmental outcomes in females.

	Cognitive (2nd Year)	Language (2nd Year)
Proportion of MOM	+15.8*	+18.77**

Note. + sign is the positive correlation, - sign is the negative correlation; *, p<0.05; **, p<0.01; ***, p<0.001; MOM, mother's own milk.

Power analysis
Power analysis was conducted using a simulation approach with linear mixed-effect models in R. The models included a random intercept for each subject. A total of 5000 simulations for each model were performed to assess the power. The analysis focused on testing the power of the proportion of MOM and feeding scales for each model. The resulting power for significant predictors ranged from 56.7% to 97.0%.
